# Study on the Mechanisms and Key Influencing Factors of Paclitaxel and Indocyanine Green Co-Loading in Lipid Nanoparticles

**DOI:** 10.3390/pharmaceutics18040505

**Published:** 2026-04-20

**Authors:** Weishen Zhong, Kai Yue, Genpei Zhang, Ziyang Hu

**Affiliations:** 1 School of Energy and Environmental Engineering, University of Science and Technology Beijing, Beijing 100083, China; d202110075@xs.useb.edu.cn (W.Z.);; 2 Shunde Graduate School, University of Science and Technology Beijing, Shunde District, Foshan 528399, China

**Keywords:** LNPs co-delivery, COSMO-RS, paclitaxel, indocyanine green, molecular dynamics simulation

## Abstract

**Background:** The reliable co-loading of paclitaxel (PTX) and indocyanine green (ICG) into a single lipid nanoparticle (LNP) enables synergistic antitumor delivery but remains challenging due to their distinct physicochemical properties. **Methods:** This study integrated COSMO-RS calculations, molecular dynamics simulations, and in vitro assays to systematically investigate the effects of lipid composition, drug modification, particle size, and solvent environment on dual-drug loading. **Results:** This work indicate that DMPS lipid membranes featuring highly polar headgroups and ordered bilayer structures stably bind both ICG and PTX, achieving drug-loading efficiencies (DLEs) of 7.2% and 5.6%, respectively. Carboxylation of PTX enhanced hydrogen bonding with DMPS, while alkyl chain modifications improved membrane insertion, though excessive chain length (e.g., C12) reduced stability due to increased flexibility. Increasing the LNP size from 50 nm to 250 nm raised the DLE of PTX from 4.7% to 8.1%, while sizes beyond 500 nm led to membrane destabilization. The use of 20 vol% ethanol increased total drug loading by 51% by disrupting the hydration shell of ICG and suppressing PTX aggregation; however, ethanol concentrations exceeding 40 vol% intensified drug–solvent competition and weakened membrane binding. **Conclusions:** This study provides a comprehensive elucidation of the multifactorial regulatory mechanisms underlying dual-drug loading in LNPs, offering a theoretical basis for the rational design of efficient co-delivery systems.

## 1. Introduction

In recent years, the combination of chemotherapy and photothermal therapy has demonstrated pronounced synergistic effects in tumor treatment, emerging as a promising strategy to overcome tumor heterogeneity and immunosuppression [1]. Paclitaxel (PTX), a well-established microtubule inhibitor, induces apoptosis by arresting cell mitosis [2], whereas indocyanine green (ICG), a hydrophilic photosensitizer, efficiently converts near-infrared light into heat to induce tumor cell thermal ablation and enhance immune activation [3]. Despite their mechanistic complementarity, the pronounced physicochemical disparity—stemming from the strong hydrophobicity of PTX and the hydrophilicity of ICG—poses substantial challenges to their stable, synchronous, and efficient co-loading within a single nanocarrier [4]. Liposomes, owing to their amphiphilic architecture and excellent biocompatibility, have been widely employed for the delivery of both hydrophilic and hydrophobic therapeutics [5]. However, in the context of PTX-ICG co-loading, liposomal systems are often limited by low encapsulation efficiency, asynchronous drug release, and inadequate membrane structural stability [6]. Consequently, the identification of key parameters governing co-loading behavior and the elucidation of the underlying thermodynamic and structural mechanisms remain central scientific challenges in the design of efficient combination therapy platforms [7].

The molecular composition of liposomes is a critical determinant of drug-loading behavior, with the charge and polarity of headgroups, in combination with hydrophobic chain characteristics, playing particularly important roles [8]. Negatively charged or highly polar phospholipids have been shown to provide additional hydrogen bonding and electrostatic interaction sites at the membrane interface, thereby enhancing the binding stability of hydrophilic drugs and reducing their propensity to resolubilize into the aqueous phase [9]. In contrast, neutral phospholipids favor the embedding of hydrophobic drugs within tightly packed hydrophobic domains, but offer limited stabilization for hydrophilic drugs [10]. The length and degree of saturation of hydrophobic chains further modulate membrane properties. Saturated long-chain lipids form membranes of high compactness and mechanical stability, although their reduced flexibility may restrict the incorporation of larger molecules; conversely, unsaturated chain lipids increase membrane fluidity and facilitate drug insertion, but excessive proportions can raise the risk of membrane leakage [11]. Cholesterol is frequently incorporated to fine-tune membrane compactness and mechanical strength where reduced permeability delays drug diffusion and improves retention [12]. Beyond carrier composition, molecular modification of the drug itself can markedly influence its localization and binding energy within the membrane. For instance, the introduction of hydrophilic moieties strengthens adsorption at the interfacial region, whereas hydrophobic chain extension facilitates deeper insertion into the membrane core, collectively modulating drug affinity within the lipid phase [13].

Liposomal geometric characteristics are known to play a pivotal role in determining drug-loading performance [14]. At smaller diameters, the higher surface-area-to-volume ratio increases the number of available membrane binding sites; however, the limited internal cavity volume restricts hydrophilic drug encapsulation [15]. Moreover, pronounced membrane structural alterations induced by high curvature reduce hydrophobic zone thickness and increase molecular packing density, thereby hindering deep embedding of hydrophobic drugs and ultimately limiting overall drug-loading capacity [16]. Increasing LNP size enlarges the internal aqueous cavity and thickens the hydrophobic bilayer region, which favors the encapsulation of hydrophilic drugs and enhances the stable association of hydrophobic drugs, leading to improved loading efficiency [17]. Nevertheless, when the diameter exceeds the micrometer scale, disordered lipid molecular arrangement may occur, potentially generating localized membrane depressions that compromise structural stability and render vesicles susceptible to aggregation, deformation, or premature drug leakage [18]. Therefore, the modulation of LNP size must strike a balance between maintaining membrane structural stability and maximizing drug-loading capacity [19].

The addition of organic solvents is widely employed to enhance liposomal drug-loading performance, acting not only by regulating drug dispersion states in solution but also by modulating loading behavior through alterations in membrane structure [20]. At low concentrations, alcohols reduce system polarity, thereby facilitating the insertion of hydrophobic drugs into the membrane and enhancing the interfacial binding potential of hydrophilic drugs [21]. However, this effect is strongly concentration-dependent. At elevated solvent proportions, drugs are preferentially retained within the solvent phase, thereby diminishing their distribution into the lipid bilayer. Concurrently, excessive alcohol disrupts lipid packing, reduces membrane thickness, and compromises the continuity of the hydrophobic domain, ultimately impairing vesicle structural stability and increasing the risk of premature drug leakage [22]. Thus, the selection of an appropriate solvent concentration is critical for determining overall loading efficiency. Despite substantial progress, most existing studies have focused on isolated factors or single drug loading, with limited attention given to the synergistic co-loading mechanisms under multifactorial coupling of lipid composition, particle size, and solvent effects.

In this study, COSMO-RS thermodynamic modeling, molecular dynamics simulations, and in vitro experimental validation are integrated to systematically evaluate the influence of lipid composition, LNP size, solvent ratio, and drug structural modification on the synergistic co-loading behavior of PTX and ICG. Drug distribution patterns within lipid bilayers of varying structural characteristics are analyzed, and the thermodynamic driving forces together with the structural regulation mechanisms governing the loading process are elucidated, thereby establishing a theoretical framework for the rational design and optimization of dual-drug LNP delivery systems.

## 2. Methods

**Figure 1:**
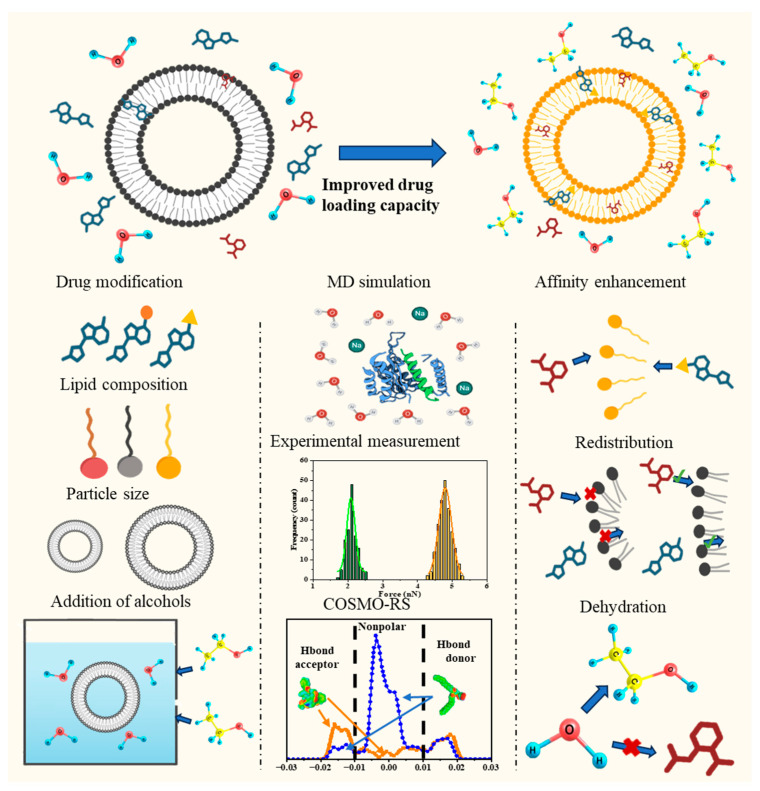
Insights into the mechanisms of enhanced LNP drug loading through integrated analyses.

Figure 1 provides a schematic overview of the methodological framework employed in this work. Four key regulatory factors, namely drug modification, lipid composition, particle size, and solvent environment, were systematically investigated through the integration of COSMO-RS calculations, molecular dynamics simulations, and experimental measurements. The figure illustrates how these approaches were combined to reveal the underlying mechanisms of enhanced LNP drug loading, including affinity enhancement, redistribution within bilayers, and solvent-induced dehydration.

### 2.1. Materials

1,2-Dimyristoyl-sn-glycero-3-phospho-L-serine (DMPS), 1,2-dimyristoyl-sn-glycero-3-phosphoglycerol (DMPG), 1,2-dimyristoyl-sn-glycero-3-phosphocholine (DMPC), lysophosphatidylcholine (LPC), indocyanine green (ICG), anhydrous ethanol, propylene glycol, uranyl acetate, and other routine analytical reagents were purchased from Macklin Biochemical Co., Ltd. (Shanghai, China). Paclitaxel (PTX) and paclitaxel fluorescein isothiocyanate conjugate (PTX-FITC) were purchased from Chongqing Qiyue Biotechnology Co., Ltd. (Chongqing, China). Ultrapure water was used throughout all experiments. Ultrafiltration centrifuge tubes with a molecular weight cut-off of 10 kDa were purchased from Thermo Fisher Scientific (Guangzhou, China) and used for purification and removal of free drugs.

### 2.2. COSMO-RS Analysis

COSMO-RS calculations were employed to predict the partitioning behavior and binding energies of indocyanine green (ICG), paclitaxel (PTX), and its modified derivatives (Paclitaxel-Succinic Acid (PTX-SA), Paclitaxel-Glutaric Acid (PTX-GA), Paclitaxel-Dodecanoic Acid (PTX-DA)) in various lipid membranes and mixed-solvent environments. The initial three-dimensional structures of the drug molecules were constructed using GaussView 6.0 [23,24], and lipid membrane structures were obtained from the CHARMM-GUI platform [25,26]. Both drug and lipid models were geometrically optimized at the BP/TZVP level [27], and COSMO-format files were generated. The σ-profiles of the drug and lipid molecules were then calculated using COSMOthermX 2019 (COSMOlogic GmbH, Leverkusen, Germany) [28] and categorized into hydrogen-bond donor regions (σ > 0.01), hydrogen-bond acceptor regions (σ < −0.01), and nonpolar regions (−0.01 ≤ σ ≤ 0.01). Corresponding σ-potential curves were further obtained for detailed energy distribution analysis.

The COSMO_mic_ module of COSMO_thermX_ was utilized to compute the partition coefficients (logP) of the drugs in lipid/water systems. Based on a stratified membrane model along the normal direction, residual chemical potentials at different embedding depths were calculated to obtain the free energy profile (ΔG), along with corresponding enthalpic (ΔH) and entropic (−TΔS) contributions, thereby elucidating the driving mechanisms of membrane embedding. In addition, a biphasic system comprising DMPS lipid membranes and alcohols (ethanol or propylene glycol) at varying volume fractions (0%, 20%, 40%, 60%, 80%, and 100%) was constructed. The logP values of the drugs in lipid/solvent systems were evaluated, and the effects of alcohols on drug dehydration and hydrogen-bond competition were systematically analyzed using σ-profile analysis.

### 2.3. MD Simulations

Four lipid bilayer models (DMPS, DMPG, DMPC, and LPC) with dimensions of 6.0 nm × 6.0 nm were constructed to investigate drug–membrane binding characteristics. A schematic illustration of the simulation systems and molecular components used in this study is provided in Figure S1. One drug molecule (ICG, PTX, or its derivatives PTX-SA, PTX-GA, PTX-DA) was inserted to simulate spontaneous membrane embedding. Each system was equilibrated for 100 ns under the NPT ensemble. Subsequently, a single drug molecule located at the binding site was extracted using steered molecular dynamics (SMD) simulations [29] at a constant pulling velocity, and the corresponding force–distance curves were recorded to determine the maximum binding force and associated energy barrier. The full molecular compositions of these atomistic bilayer systems, including lipid, drug, water, and ion numbers, are summarized in Table S1, and representative initial and final configurations are provided in Figure S2.

DMPS lipid vesicles with diameters of 50, 100, 250, and 500 nm were constructed to evaluate the effect of particle size on drug transmembrane permeation. A single ICG or PTX molecule was added to each system, and the Martini coarse-grained model was employed. Constant-velocity pulling (velocity: 0.01 nm/ps; spring constant: 1000 kJ/mol·nm^2^) was applied to simulate the permeation process, and average transmembrane forces along with energy barriers were calculated. The detailed compositions of the coarse-grained membrane models used for particle size analysis are listed in Table S2, and representative initial and final configurations are shown in Figure S3.

A 100 nm DMPS lipid vesicle was used to examine solvent-mediated effects on drug binding. Systems containing a single ICG or PTX molecule were simulated in ethanol–water or propylene glycol–water mixtures at volume fractions of 0%, 20%, 40%, 60%, 80%, and 100%. Each system was simulated for 100 ns, and hydrogen-bond counts along with electrostatic and van der Waals interaction energies between the drug and membrane were analyzed. The molecular compositions of the atomistic solvent-containing systems used for solvent-effect analysis are summarized in Table S3.

All atomistic simulations used the CHARMM36 force field for lipids, while drug parameters were generated using the GAFF force field with RESP charges. Systems were solvated in a 6.1 × 6.1 × 6.1 nm^3^ TIP3P water box, and 0.15 M NaCl was added to ensure charge neutrality. Temperature (310 K) and pressure (1 bar) were maintained using a Langevin thermostat and Parrinello–Rahman barostat [30]. Electrostatic interactions were calculated with the particle mesh Ewald (PME) method [31], and van der Waals interactions were truncated at 1.2 nm. A time step of 2 fs was employed for integration, and each simulation was performed for 100 ns. Steered molecular dynamics pulling simulations were carried out using the GROMACS pull code with a pulling velocity of 0.01 nm/ps and a spring constant of 1000 kJ/mol·nm^2^. Coarse-grained vesicle simulations utilized the Martini 2.2 model, with box dimensions and solvent numbers adjusted according to vesicle size. Post-processing was conducted using GROMACS 2018 [32] and VMD [33].

### 2.4. In Vitro Experiments

DMPS lipid was dissolved in anhydrous ethanol to a final lipid concentration of 12 mM. Ultrapure water was applied as the aqueous phase. A dual-channel microfluidic pump (Dolomite Microfluidics, Royston, UK) was employed to inject the lipid–ethanol solution and aqueous phase into a Y-shaped microfluidic chip for mixing. Total flow rates of 6, 12, 3, and 24 mL/min were used to prepare LNP suspensions with desired particle sizes. Solvent exchange during mixing induced spontaneous lipid self-assembly into vesicles. The resulting vesicle suspensions were transferred into 10 kDa MWCO ultrafiltration centrifuge tubes and centrifuged at 3000 rpm for 30 min to remove residual organic solvents and impurities, yielding purified LNP vesicles. DMPC, DMPG, and LPC LNPs were prepared in a similar manner at a total flow rate of 6 mL/min. All LNP suspensions were stored at 4 °C until subsequent drug loading and characterization. For lipid-type comparison, DMPC-, DMPG-, and LPC-based LNPs were also prepared and characterized in vitro. However, DMPS was selected as the primary experimental system for the subsequent particle size and solvent-fraction studies because it showed the most favorable loading behavior in the preliminary comparative analyses.

LNP morphology was examined by transmission electron microscopy (TEM). Briefly, 5 μL of LNP suspension was deposited onto a carbon-coated copper grid and allowed to adsorb for 5 min. Excess liquid was removed with filter paper, followed by negative staining with 2% uranyl acetate for 2 min to enhance membrane contrast. The samples were dried under vacuum and imaged using a TEM (Tecnai G2 20 S-TWIN, FEI, Hillsboro, OR, USA). Particle sizes were determined by dynamic light scattering (DLS) with a Malvern Zetasizer (Malvern Panalytical Ltd., Malvern, Worcestershire, UK). DLS measurements were used to characterize the size distributions of the LNP formulations prepared under different microfluidic conditions.

Purified DMPS LNPs were dispersed in aqueous solutions containing different alcohol volume fractions (0%, 20%, 40%, 60%, 80%, and 100%), with the total lipid concentration adjusted to 12 mM. ICG or PTX-FITC was added at a drug-to-lipid mass ratio of 20%, and the mixture was incubated at 37 °C for 1 h. After incubation, vesicle suspensions were transferred to 10 kDa MWCO ultrafiltration centrifuge tubes and centrifuged at 3000 rpm for 10 min to remove unencapsulated drugs, yielding drug-loaded LNPs. Drug-loading efficiencies for ICG and PTX-FITC were adequate using a microplate reader at excitation wavelengths of 785 nm and 494 nm, respectively.

Drug–LNP interaction forces were measured using an atomic force microscope (AFM, MFP3D-Bio, Oxford Instruments, Santa Barbara, CA, USA). AFM probes were dip-coated in a 6 mM drug solution for 3–5 min, and the coating procedure was repeated at least five times to ensure adequate surface modification. LNP suspensions were deposited onto a culture dish substrate for immobilization. AFM measurements were performed under liquid conditions. For force–distance curve acquisition, the loading force was set to 5 nN, the working distance to 1 μm, and the approach speed to 5 μm/s. For each sample, 150 independent force curves were collected at different substrate positions, and the binding force distributions were obtained from the statistical analysis of these curves. The average binding force was then calculated to reduce measurement variability.

## 3. Results and Discussion

### 3.1. Influence of Lipid Polarity on Drug Loading

**Figure 2:**
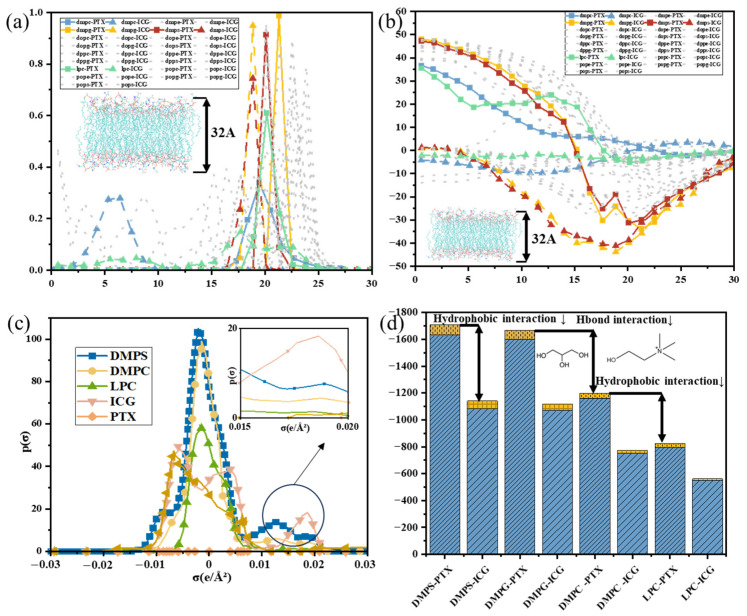

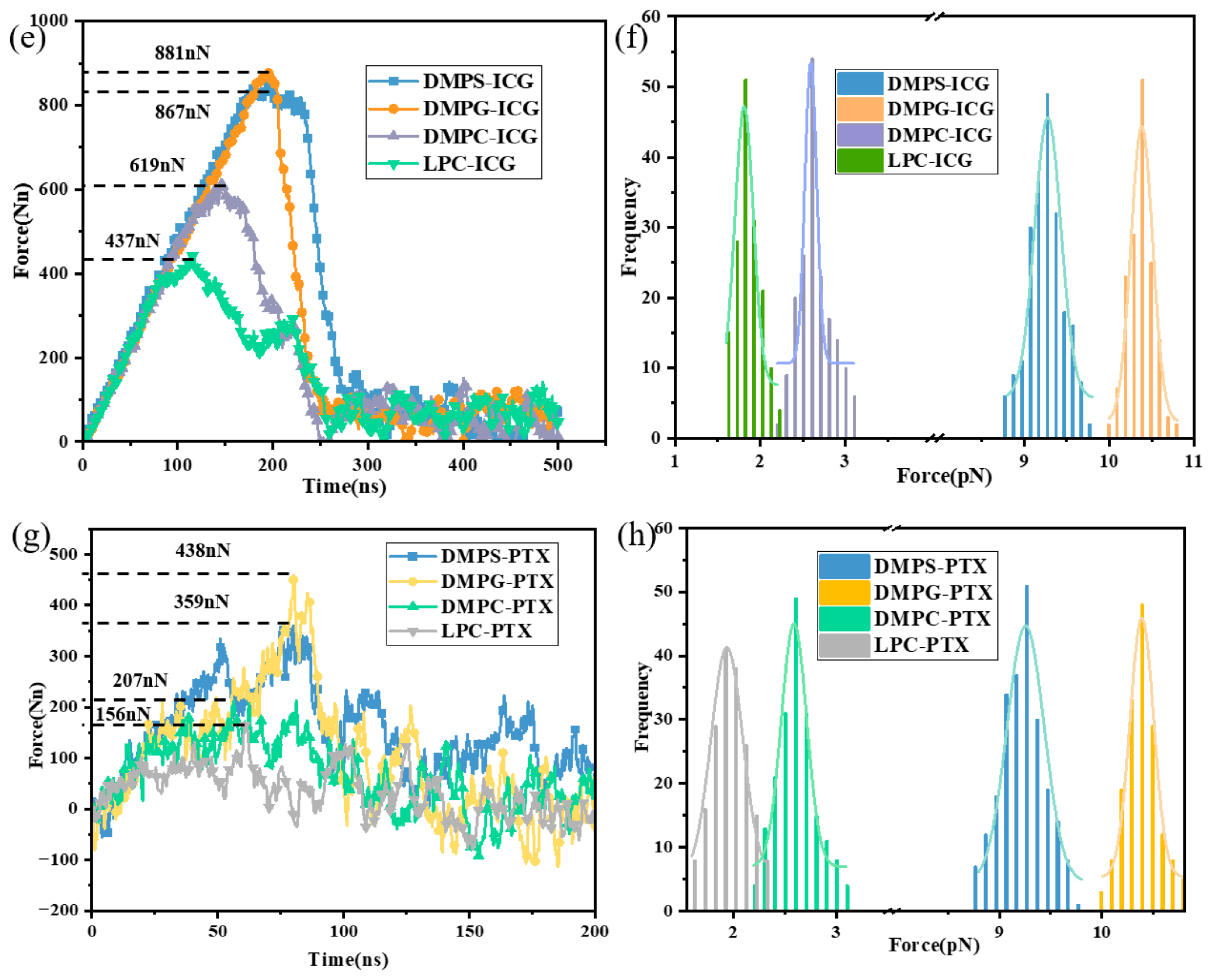
Analysis of ICG and PTX loading in different lipid systems. (**a**) Probability distribution of drug embedding depth in different lipid membranes; (**b**) system free energy distribution at corresponding depth positions; (**c**) σ-profile plots of each component, reflecting their distribution in polar regions; (**d**) interaction energy (van der Waals and Coulombic) between drugs and four lipid membranes; (**e**,**f**) maximum force–time curve during pulling simulation of drug detachment from the lipid surface; (**g**,**h**) distributions of ICG binding force with different lipids measured by AFM.

The loading stability of the hydrophilic photosensitizer ICG is primarily governed by the polarity of lipid headgroups and the hydrogen bonding network. The COSMO-RS calculations (Figure 2a) revealed that the thermodynamically optimal binding site of ICG in DMPS and DMPG membranes is located at the headgroup–water interface (approximately 25 Å from the membrane surface), where the system exhibits the lowest free energy (ΔG = −35 kJ/mol, Figure 2b). At this interface, enthalpic contributions (ΔH) dominate the binding process (Figure S4). The sulfonate and amide groups of ICG form multiple stable hydrogen bonds with phosphate moieties in DMPS/DMPG, conferring strong binding affinity. At this interface, enthalpic contributions (ΔH) dominate the binding process (Figure S4). The sulfonate and amide groups of ICG form multiple stable hydrogen bonds with phosphate moieties in DMPS/DMPG, conferring strong binding affinity, respectively, indicating significantly weaker binding stability. Partition coefficient (logP) values further support this trend. In contrast, for DMPC and LPC, ICG binding sites also localize near the interface, but the corresponding free energies increase to −1 kJ/mol and +2 kJ/mol, respectively, indicating significantly reduced binding stability. Partition coefficient (logP) values further support this trend, where logP values for ICG in DMPS/water and DMPG/water systems are 24 and 23, respectively, while values for DMPC/water and LPC/water systems are both below 10 (Figure S5).

The σ-profile analysis (Figure 2c) demonstrated that DMPS and DMPG exhibit dense hydrogen-bond donor regions (σ > 0.01 eÅ^−2^), which form complementary hydrogen bonds with acceptor regions of ICG, thereby enhancing binding stability at the membrane surface. Conversely, DMPC and LPC exhibit weaker polar headgroups with limited hydrogen bonding capacity, and the single-chain structure of LPC results in a loose surface, significantly reducing binding efficiency. Molecular dynamics (MD) simulations further validated these conclusions. As shown in Figure 2d, the total interaction energy between ICG and the DMPS membrane is −142 kJ/mol, with hydrogen bonding and hydrophobic interactions contributing >90%. The average number of hydrogen bonds formed is 4–5, higher than that in DMPC and LPC systems (Figure S6). Steered MD simulations revealed that the maximum unbinding force required to detach ICG from DMPS is approximately 867 nN, while forces decrease to 619 nN and 437 nN for DMPC and LPC, respectively (Figure 2e). Experimental observations were consistent with computational predictions (Figure S7), AFM measurements indicated stronger binding forces for ICG–DMPS interactions than those for other lipid systems (Figure 2f). In summary, stable loading of ICG relies on a hydrogen bonding network constructed by sulfonate and amide groups. DMPS and DMPG provide optimal binding environments due to their strongly polar headgroups and ordered double-chain structures. In contrast, weak polarity and loose packing in DMPC and LPC compromise loading efficacy. Thus, hydrophilic drug formulations should preferentially employ lipids with strong polarity and well-ordered double-chain architectures to ensure stable loading and minimize dissociation risk.

As a typical hydrophobic chemotherapeutic agent, PTX binding to lipid membranes is dominated by hydrophobic interactions, supplemented by hydrogen bonding [34]. COSMO-RS results indicated that the optimal binding site of PTX in DMPS membranes is located within the hydrophobic tail region (~25 Å from the surface), with a free energy of −45 kJ/mol. For DMPC and LPC, binding free energies are only −12 kJ/mol and −8 kJ/mol, respectively. Additionally, the logP of PTX in DMPS/water systems is 27, significantly higher than values in DMPC/water and LPC/water (Figure S8), confirming a stronger affinity between DMPS and PTX. σ-profile analysis revealed significant overlap between nonpolar regions of DMPS and aromatic rings/t-butyl groups of PTX, indicating strong van der Waals interactions. Concurrently, phosphate groups in DMPS form 2–3 hydrogen bonds with hydroxyl groups of PTX, reducing dehydration energy barriers and enhancing embedding depth (Figure S9). Although DMPC possesses a dense hydrophobic core, its insufficiently polar headgroups limit PTX binding to the interface. The single-chain structure of LPC reduces membrane thickness and compromises hydrophobic continuity, preventing stable PTX accommodation. Energy calculations support these findings: van der Waals interaction energy for PTX-DMPS is −165 kJ/mol, while values for PTX-DMPC and PTX-LPC decrease to −92 kJ/mol and −78 kJ/mol, respectively. Steered simulations showed that the force required to detach PTX from DMPS is 438 nN, compared to 207 nN and 56 nN for DMPC and LPC. In vitro experiments validated these trends. PTX loading efficiency in DMPS LNPs is significantly higher than in DMPC/LPC systems (Figure S10), and DMPS-PTX binding forces exceed those of other lipid systems. Together with the stronger ICG-DMPS interactions observed in the comparative experiments, these results indicate that DMPS provides the most favorable loading behavior among the tested lipid formulations and was therefore selected for the subsequent size- and solvent-dependent studies. Therefore, efficient PTX embedding requires both a continuous, ordered hydrophobic core to maximize van der Waals interactions and polar headgroups to provide auxiliary hydrogen bonds that reduce entry barriers. DMPS fulfills both criteria and is the optimal carrier, whereas DMPC’s polarity deficiency and LPC’s structural instability severely limit loading capacity.

**Figure 3:**
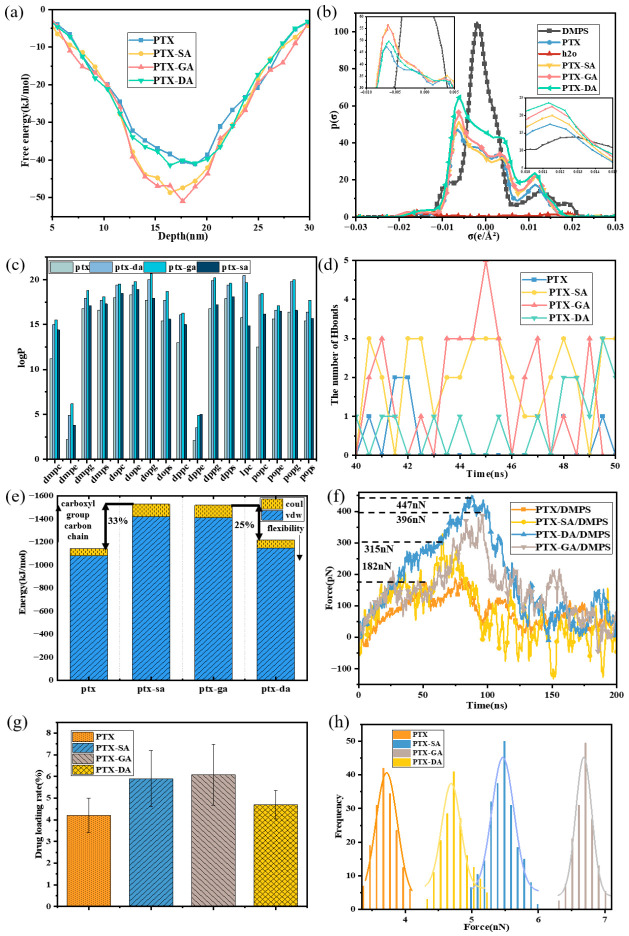
Analysis of the effect of carboxyl modification on PTX loading in DMPS. (**a**) System free energy distribution of PTX and its derivatives at different embedding depths in DMPS; (**b**) σ-profile plots of each component, reflecting their distribution in polar regions; (**c**) partition coefficients (logP) of different PTX derivatives in various lipid/water systems; (**d**) the number of hydrogen bonds between PTX derivatives and DMPS; (**e**) interaction energy (van der Waals and Coulombic) between PTX derivatives and DMPS; (**f**) maximum detachment force of PTX derivatives from DMPS in pulling simulations; (**g**) loading efficiency of PTX and its derivatives in DMPS LNPs measured by microplate reader; (**h**) distributions of binding force between PTX derivatives and DMPS measured by AFM.

Carboxyl and carbon chain modifications significantly improve the membrane affinity of PTX compared with unmodified PTX. Binding free energy for PTX-SA with DMPS increases by ~12% (Figure 3a) primarily due to added hydrogen-bond donors from carboxyl groups and enhanced hydrophobic embedding via short-chain structures (Figure 3b). The carbon chain is extended to GA (PTX-GA) and hydrophobicity further increases. The hydrogen-bond counts between PTX and DMPS rise from 1 to 3 (Figure 3c), and logP in DMPS/water increases from 31 to 37 (Figure 3d). However, when the chain length reaches DA (PTX-DA), binding free energy slightly decreases, and logP declines. This phenomenon is attributed to increased conformational flexibility from excessively long side chains, which reduces hydrophobic matching efficiency and effective embedding space utilization. MD simulations yielded similar conclusions, van der Waals interaction energies for PTX-SA-DMPS and PTX-GA-DMPS are −178 kJ/mol and −191 kJ/mol, respectively, both higher than that for PTX-DA-DMPS (Figure 3e). As shown in Figure 3f, the detachment force for PTX-GA (493 pN) is significantly higher than for PTX-DA (329 pN). In vitro experiments further confirmed this trend; both loading efficiency and binding force for PTX-DA in DMPS are lower than those for PTX-GA (Figure 3g,h). In conclusion, carboxyl modifications enhance PTX’s membrane affinity through dual mechanisms introducing polar groups and hydrophobic chains. Moderate chain lengths synergistically improve hydrogen bonding and hydrophobic interactions, but excessive chain lengths reduce binding site stability due to increased molecular flexibility, ultimately limiting loading efficiency.

### 3.2. Influence of LNP Size on Drug-Loading Behavior

**Figure 4:**
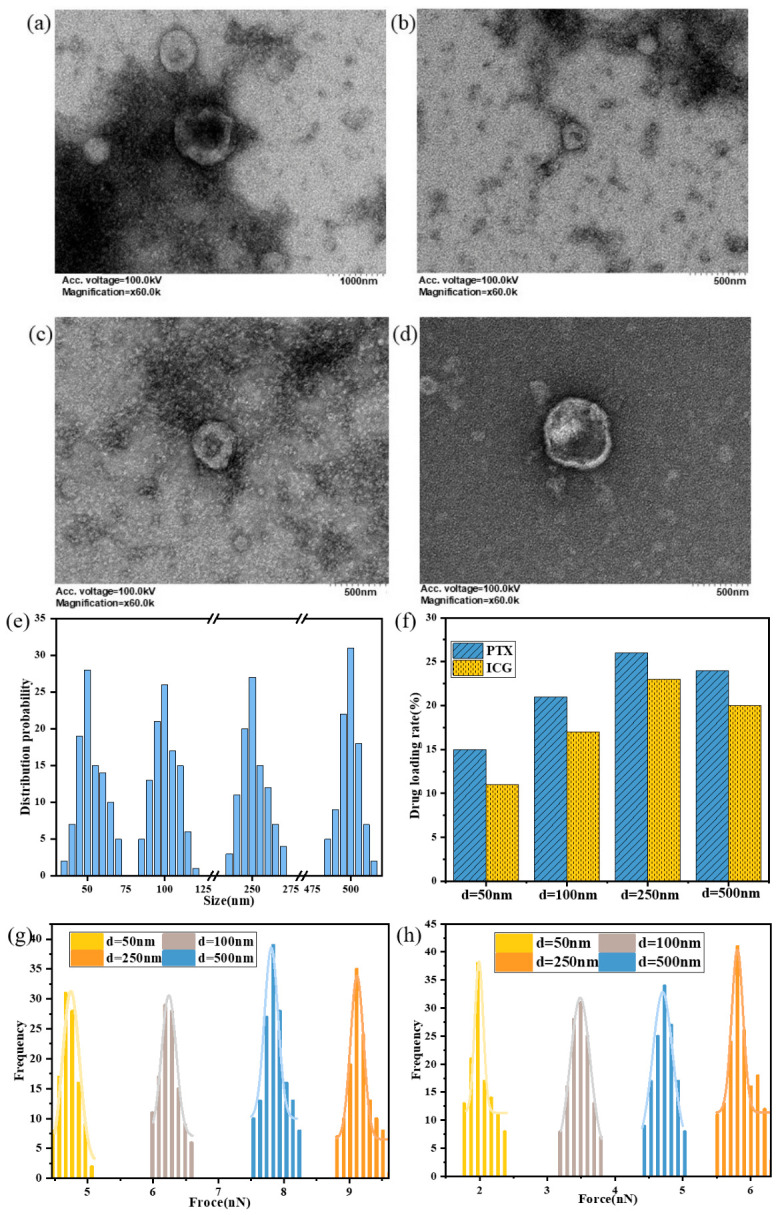
Effect of LNP size on loading of PTX and ICG. (**a**–**d**) TEM images of DMPS LNPs with different particle sizes; (**e**) particle size distribution of lipids; (**f**) the drug-loading rate of PTX and ICG in LNPs with different particle sizes; (**g**) ICG-DMPS binding force distribution frequency; (**h**) PTX-DMPS binding force distribution frequency.

Based on the comparative lipid-type analysis presented above, DMPS was selected as the representative experimental system for the size-dependent study because it showed the most favorable loading behavior for both ICG and PTX. Accordingly, this study systematically investigated the size-dependent loading behavior of hydrophilic photosensitizer ICG and hydrophobic chemotherapeutic drug PTX by preparing four DMPS LNP formulations with different particle sizes (50 nm, 100 nm, 250 nm, and 500 nm). LNP size is a critical physical parameter that directly influences molecular packing density and drug diffusion barriers, thereby determining the spatial distribution and binding stability of drugs within the membrane. As shown in Figure 4a–e, dynamic light scattering (DLS) and transmission electron microscopy (TEM) characterization confirmed that all LNPs exhibited monodisperse spherical morphology with size deviations < 5%, meeting high-precision experimental requirements.

Microplate reader quantification (Figure 4f) revealed that when LNP size was below 250 nm, ICG loading efficiency increased linearly from 3.4% to 6.1% and PTX from 4.7% to 8.1% with increasing size. This positive correlation arose from reduced membrane curvature, where decreased curvature minimized lipid molecular tilt angles, resulting in more ordered hydrophobic packing and providing a stable microenvironment for drug embedding. However, when size exceeded 500 nm, loading efficiency improvement slowed significantly, indicating a size-effect threshold potentially related to enhanced membrane fluctuations caused by declining bending rigidity. AFM force spectroscopy further validated these results; as size increased from 50 nm to 250 nm, ICG-DMPS binding forces rose from 4.7 nN to 9.5 nN and PTX-DMPS from 2.1 nN to 5.9 nN. These values were obtained from the statistical analysis of 150 independent force–distance curves for each sample, and the corresponding distributions are shown in Figure 4g,h. To further interpret the experimentally observed size-dependent loading behavior, coarse-grained membrane models with curvatures corresponding to DMPS vesicles of different equivalent diameters were used to analyze drug permeation, binding force evolution, and membrane structural perturbation.

**Figure 5:**
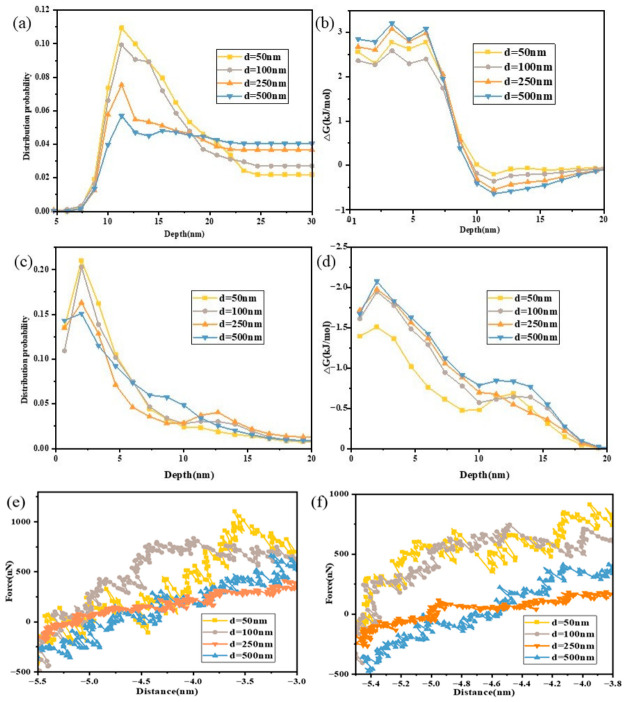
Molecular simulation analysis of PTX and ICG loading in liposomes of different particle sizes. (**a**) PTX penetration depth distribution; (**b**) free energy distribution corresponding to PTX depth; (**c**) free energy distribution corresponding to ICG depth; (**d**) ICG penetration-depth distribution; (**e**) binding force between DMPS and PTX calculated by simulation; (**f**) binding force between DMPS and ICG calculated by simulation.

The force enhancement followed a negative exponential relationship with curvature reduction, confirming that low-curvature membranes strengthen drug–lipid interfacial affinity. COSMO-RS simulations elucidated the regulatory effect of LNP size on PTX and ICG distribution. PTX exhibited a unimodal distribution in DMPS LNPs, with its probability gradually shifting from the polar headgroup region (~10–15 Å) to the membrane core (25–40 Å) as size increased (Figure 5a), indicating that larger systems favor deep PTX embedding. Lower free energy during PTX embedding in large-sized membrane cores (Figure 5b) demonstrated enhanced lipophilicity. ICG binding free energy at the membrane surface (~12–15 Å) slightly increased with size (Figure 5c), suggesting improved stability at polar headgroup regions. However, ICG binding probability remained low without significant enrichment in deep membrane regions (>20 Å) (Figure 5d), confirming its molecular structure is unsuitable for deep penetration. A secondary binding peak near the membrane surface may reflect local embedding or shallow permeation tendencies, attributable to ICG’s dense polar groups and strong hydration, which anchor it to hydrophilic headgroups through hydrogen bonding networks.

**Figure 6:**
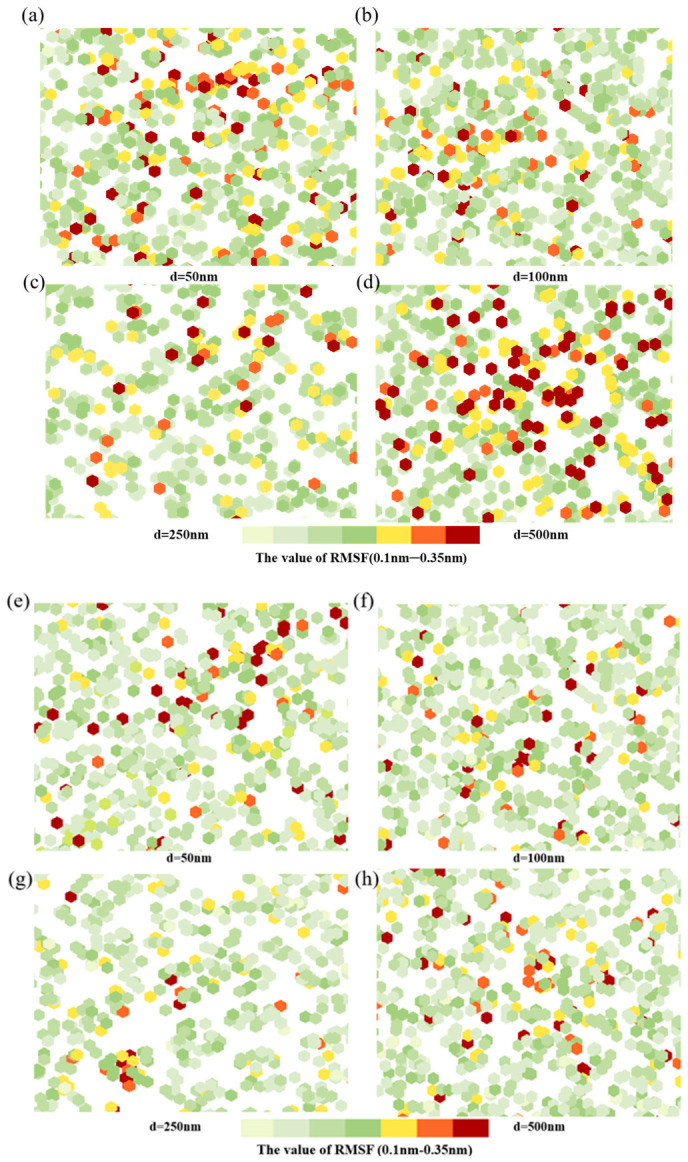
RMSF maps of lipid bilayers after drug translocation. (**a**–**d**) The RMSF of LNPs with different sizes after ICG across liposomes; (**e**–**h**) the RMSF of LNPs with different sizes after PTX across liposomes. Colors indicate lipid-atom RMSF (0.10–0.35 nm).

As shown in Figures S11 and S12, partition coefficients (logP) for ICG and PTX in DMPS/water systems increased progressively with increasing LNP size, indicating that larger vesicles promote drug transfer into the membrane phase. Steered simulations showed transmembrane forces declined markedly. The binding force between DMPS and ICG decreased from 420 nN to 260 nN and the binding force between DMPS and ICG decreased from 530 nN to 310 nN (Figure 5e,f), reflecting more stable binding in larger membranes. This trend further verifies that larger LNPs provide more embedding space and lower de-embedding energy barriers, enhancing transmembrane capability and retention stability for hydrophobic drugs. However, when the vesicle size exceeds 2500 nm, the drug retention capacity decreases. Two-dimensional membrane order parameter distributions (Figure 6) showed that as LNP size increased from 50 nm to 250 nm, structural perturbations decreased while overall order parameters increased (with disturbances concentrated centrally), indicating larger sizes help maintain membrane structural order. However, in 500 nm systems, perturbations extended throughout the membrane thickness with decreased edge order parameters, suggesting reduced membrane stability beyond 500 nm may compromise local structural integrity required for drug permeation and weaken loading capacity. These observations can be explained by curvature effects. Increasing LNP size reduces curvature, expanding polar headgroup spacing while compressing hydrophobic tail packing in small, high-curvature vesicles, producing irregular hydrophobic cores that hinder stable accommodation of bulky molecules such as PTX. In contrast, large, low-curvature LNPs approach a near-planar geometry with uniformly packed hydrophobic cores and expanded free volume, facilitating deeper PTX insertion. For ICG, binding sites remain primarily at the polar membrane surface; in high-curvature systems, enlarged headgroup spacing reduces polar group density and limits stable hydrogen-bond formation. Reduced curvature, by contrast, promotes compact headgroup packing and higher hydrogen-bond acceptor density, thereby strengthening ICG binding. However, when the curvature becomes excessively low, the surface stress of the particles decreases, leading to reduced resistance to fluid shear forces. Consequently, membrane stability is compromised, thereby limiting drug-loading capacity.

### 3.3. Influence of Different Solvents

**Figure 7:**
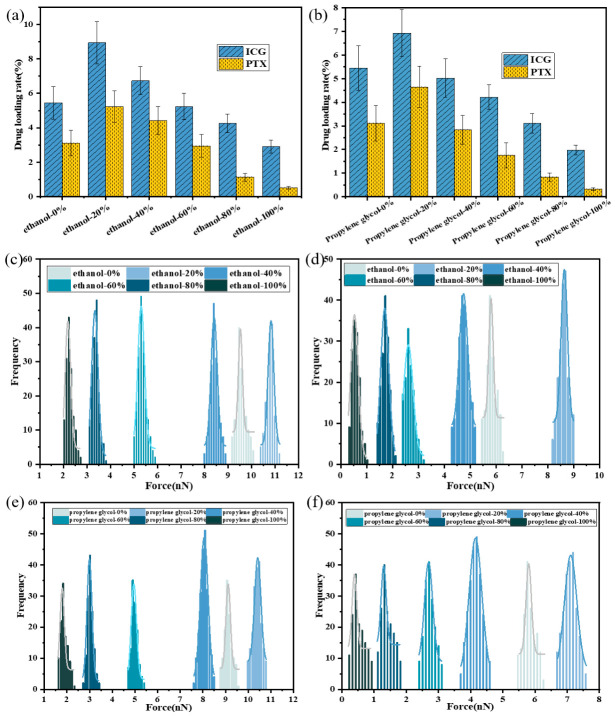
Effect of alcohol volume fraction on drug loading in DMPS LNPs. (**a**) The drug-loading rate of ICG and PTX loading in ethanol; (**b**) the drug-loading rate of ICG and PTX loading in propylene glycol; (**c**) the binding force of ICG-DMPS in ethanol; (**d**) the binding force of PTX-DMPS in ethanol; (**e**) the binding force of ICG-DMPS in propylene glycol; (**f**) the binding force of PTX-DMPS in propylene glycol.

Because DMPS exhibited the most favorable loading behavior in the preceding comparative analyses, this section further examined the regulatory effects of two common alcoholic solvents (ethanol and propylene glycol) at varying volume fractions (0%, 20%, 40%, 60%, 80%, and 100%) on drug-loading behavior in DMPS LNPs. In vitro loading assays (Figure 7a) showed that in pure aqueous systems, the loading efficiencies of ICG and PTX were 8.9% and 5.2%, respectively. The addition of low concentrations of ethanol markedly enhanced loading of both ICG and PTX. However, with further increases in ethanol content, drug loading gradually declined. Propylene glycol exhibited a similar pattern, PTX and ICG loading first increased and then decreased with rising concentrations. Notably, the maximum loading capacity in propylene glycol systems appeared at lower solvent fractions and was smaller than that observed in ethanol systems (Figure 7b). Consistent results were obtained by AFM force spectroscopy (Figure 7c,d). The addition of 20% ethanol increased the adhesion force between ICG and DMPS from 8.5 pN to 10.8 pN and between PTX and DMPS from 8.1 pN to 10.5 pN. At higher ethanol concentrations, however, adhesion forces decreased progressively, reaching 1.9 pN for ICG-DMPS and 2.3 pN for PTX–DMPS. Systems containing propylene glycol displayed comparable trends (Figure 7e,f).

**Figure 8:**
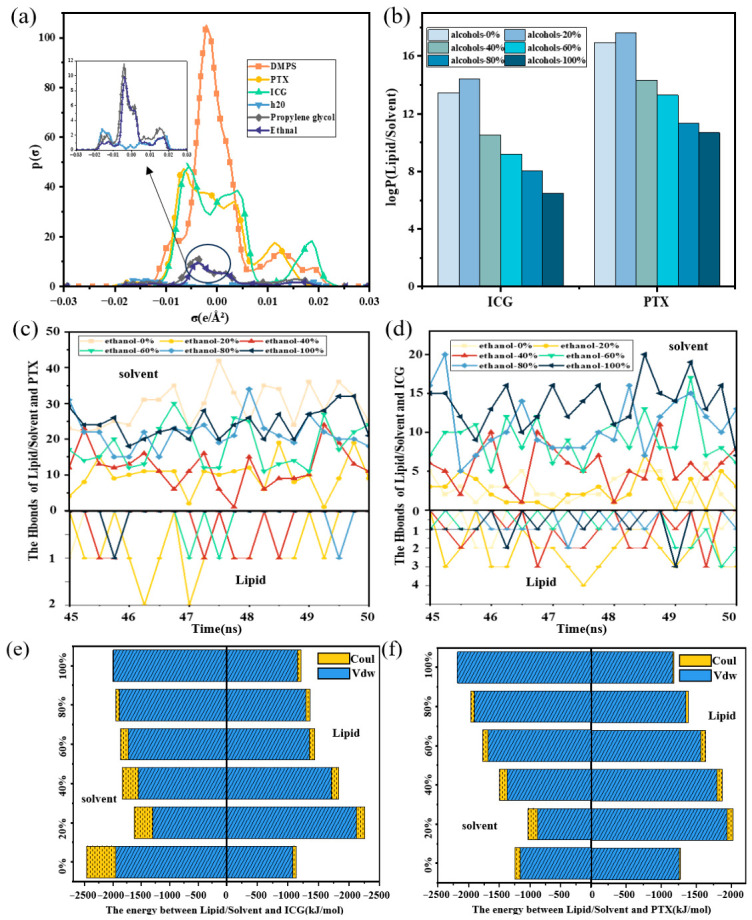
Effect of ethanol concentration on drug interactions. (**a**) σ-profile plots of each component, reflecting their distribution in polar regions; (**b**) logP variation in ICG/PTX in water/ethanol mixtures; (**c**) the number of H-bonds between ICG and LNPs/solvent; (**d**) the number of H-bonds between PTX and LNPs/solvent; (**e**) the interaction energy between ICG and LNPs/solvent; (**f**) the interaction energy between PTX and LNPs/solvent.

COSMO-RS theoretical analysis was employed to elucidate how changes in solvent polarity regulate drug-loading behavior. As shown in Figure 8a ICG molecules possess densely distributed polar groups, with pronounced peaks in the σ < −0.01 (hydrogen-bond donor) and σ > 0.01 (hydrogen-bond acceptor) regions. This polarity enables ICG to establish stable hydrogen bonding networks with water in pure aqueous systems, resulting in strong hydration that imposes a high desolations barrier. At low ethanol concentrations, ethanol molecules form hydrogen bonds with water, weakening water–ICG interactions and thereby reducing the dehydration barrier, which facilitates ICG partitioning into the lipid membrane. However, at higher ethanol fractions, the increased nonpolar character of the medium drives ICG’s nonpolar fragments to preferentially associate with ethanol through hydrophobic interactions, leading to its retention in the solvent phase and a reduced partition coefficient in DMPS/solvent systems (Figure 8b). In addition, ICG loading behavior is jointly determined by solvent polarity and its molecular architecture. Although low concentrations of alcohol facilitate ICG dehydration and promote adsorption at the membrane surface, its rigid polycyclic hydrophobic backbone is sterically incompatible with the tightly packed acyl chains of the membrane core. Consequently, high alcohol concentrations cannot promote deeper transmembrane penetration. By contrast, PTX exhibits weaker polarity, with electron density predominantly distributed in the −0.01 ≤ σ ≤ 0.01 (nonpolar) region. In pure aqueous solutions, PTX tends to self-aggregate into hydrophobic clusters with low dispersibility, limiting effective contact with liposomes. The introduction of moderate ethanol levels increases solvent nonpolarity, enhancing PTX dispersibility and its probability of membrane contact, thereby facilitating insertion into the hydrophobic core and stabilizing binding via van der Waals interactions. At high ethanol concentrations, however, strengthened PTX–solvent van der Waals interactions favor PTX solubilization in the solvent phase while disfavoring membrane binding, ultimately decreasing the partition coefficient in DMPS/solvent systems.

Furthermore, hydrogen-bond counts and interaction energies between drugs, solvents, and DMPS were statistically analyzed across different solvent systems. As shown in Figure 8c,d in systems containing 20% ethanol, ICG formed the fewest hydrogen bonds with water but the most with DMPS. Relative to pure aqueous systems, the van der Waals energy between DMPS and ICG increased from −1249 to −1783 kcal mol^−1^, and electrostatic energy strengthened to −125 kcal mol^−1^, while the van der Waals energy between DMPS and water decreased to −806 kcal mol^−1^. With further increases in ethanol concentration, however, both the hydrogen-bond numbers and interaction energies between ICG and DMPS decreased substantially. PTX, by contrast, relied predominantly on nonpolar interactions. When the ethanol fraction increased to 20%, the van der Waals energy between PTX and DMPS rose markedly from −1109 to −2347 kcal mol^−1^, while hydrogen-bond counts remained nearly unchanged, indicating that hydrophobic interactions are the primary driving force for PTX binding. At high ethanol concentrations (Figure 8e,f), PTX could still associate with DMPS, but the van der Waals interaction energy between them decreased significantly, whereas the van der Waals energy between PTX and the solvent increased. This shift highlights strengthened PTX–solvent association and reduced effective interaction with the membrane.

When comparing solvent types, propylene glycol exhibits a high density of both nonpolar and polar regions (Figure 8), which enables it to strongly perturb hydration networks at low concentrations and thereby promote drug dehydration. However, its molecular structure, characterized by longer carbon chains and larger nonpolar domains, facilitates the formation of stable drug–solvent complexes at higher concentrations. This effect is particularly pronounced for the hydrophobic drug PTX, where such associations inhibit membrane binding and ultimately result in lower maximum loading in DMPS/propylene glycol systems. In summary, alcoholic solvents regulate drug loading primarily by modulating hydrogen bonding and nonpolar interactions at both drug–solvent and drug–membrane interfaces. At low concentrations, alcohol molecules effectively disrupt hydration shells, increasing the probability of drug transfer to the membrane phase. At high concentrations, however, drugs preferentially associate with the solvent, reducing membrane affinity and thereby decreasing lipid loading capacity. Furthermore, differences in alcohol polarity contribute to solvent-dependent variations in drug-loading efficiency within lipid membranes.

## 4. Conclusions

This study systematically deciphered the effects of lipid composition, particle size, and solvent environment on the loading performance of ICG, PTX, and its carboxyl-modified derivatives through integrated COSMO-RS calculations, molecular dynamics simulations, and in vitro experiments. Lipid composition was found to determine drug binding sites and dominant interaction modes. DMPS, with strongly polar headgroups and an ordered double-chain structure, provided the most favorable binding environment for ICG (hydrogen bond-dominated) and PTX (van der Waals-dominated), achieving the highest loading efficiency. Carboxyl modification significantly enhanced PTX–membrane affinity, with PTX-DA exhibiting the most negative binding energy and the highest loading performance. However, excessively long alkyl chains did not yield further improvements due to increased molecular flexibility. The influence of size on drug loading was nonlinear. Within the 50–250 nm range, increasing particle size reduced membrane curvature and transmembrane energy barriers, thereby enhancing embedding stability. Beyond 500 nm, decreased membrane structural stability led to saturation of loading efficiency. Solvent effects were drug-dependent. For ICG, alcohols disrupted the drug–water hydrogen-bond network, reducing hydration energy and promoting transfer into the lipid bilayer. For PTX, moderate reductions in solvent polarity improved dispersibility, minimized hydrophobic aggregation, and increased membrane contact probability. For both drugs, loading efficiency reached a maximum at ~20% (*v*/*v*) alcohol, with higher concentrations decreasing loading due to enhanced drug–solvent hydrophobic association. Ethanol induced weaker perturbations of membrane structure and exhibited a broader optimal concentration range, whereas propylene glycol improved PTX dispersibility at low concentrations but promoted drug retention in the solvent phase at high concentrations. LNP drug loading is synergistically regulated by lipid polarity, side-chain modification, particle size control (optimal ~100 nm), and solvent composition (optimal ~20% *v*/*v* alcohol). These findings offer a theoretical framework for the structural optimization of lipid carriers and solvent strategies in the rational design of dual-drug co-delivery systems. It should be noted that the present study focused on the loading behavior of drug-loaded LNPs and the mechanistic effects of lipid composition, particle size, solvent environment, and drug structural modification. A systematic evaluation of the stability time or disintegration behavior of drug-loaded LNPs under physiological aqueous conditions was not included in this work and should be addressed in future studies.

## Data Availability

The data supporting the findings of this study are available from the corresponding author upon reasonable request.

## Supplementary Materials

The following supporting information can be downloaded at: https://www.mdpi.com/article/10.3390/pharmaceutics18040505/s1, Figure S1. Schematic illustration of the simulation systems and molecular components used in this study, Figure S2. Representative initial and final configurations of an atomistic bilayer system used in this study, Figure S3. Representative initial and final configurations of the coarse-grained membrane model used for particle-size analysis. The model represents a local membrane region with curvature corresponding to a vesicle of the indicated equivalent diameter, Figure S4. System enthalpy distribution at corresponding depth positions, Figure S5. Partition coefficient (logP) of ICG in different lipid/water systems, Figure S6. The number of H-bonds between DMPS and ICG, Figure S7. The drug- loading rate of ICG, Figure S8. Partition coefficient (logP) of PTX in different lipid/water systems, Figure S9. The number of H-bonds between DMPS and PTX, Figure S10. The drug- loading rate of PTX, Figure S11. Partition coefficient (logP) of PTX, Figure S12. Partition coefficient (logP) of ICG, Table S1. Molecular compositions of the atomistic bilayer systems used in this study, Table S2. Molecular compositions of the coarse-grained membrane models used for particle -size analysis, Table S3. Molecular compositions of the atomistic solvent-containing systems used for solvent-effect analysis
