# Supplementary material for: Study on the Mechanisms and Key Influencing Factors of Paclitaxel and Indocyanine Green Co-Loading in Lipid Nanoparticles
Source: Pharmaceutics. 2026 Apr 20;18(4):505. doi: 10.3390/pharmaceutics18040505 (PMC13119261; doi:10.3390/pharmaceutics18040505)
Supplement: Supplementary file 1 [file pharmaceutics-18-00505-s001.zip › pharmaceutics-4239113-supplementary.pdf]

Study on the mechanisms and key influencing factors of liposomal co-loading of paclitaxel and indocyanine green

*Weishen Zhong[1)], Genpei Zhang[1)], Ziyang Hu[1,2)], Kai Yue[1,2) *]*

1) Shunde Graduate School of University of Science and Technology Beijing, Shunde, Guangdong Province 528399, China

2) School of Energy and Environmental Engineering, University of Science and Technology Beijing, Beijing 100083, China

**Corresponding author: yuekai@ustb.edu.cn*

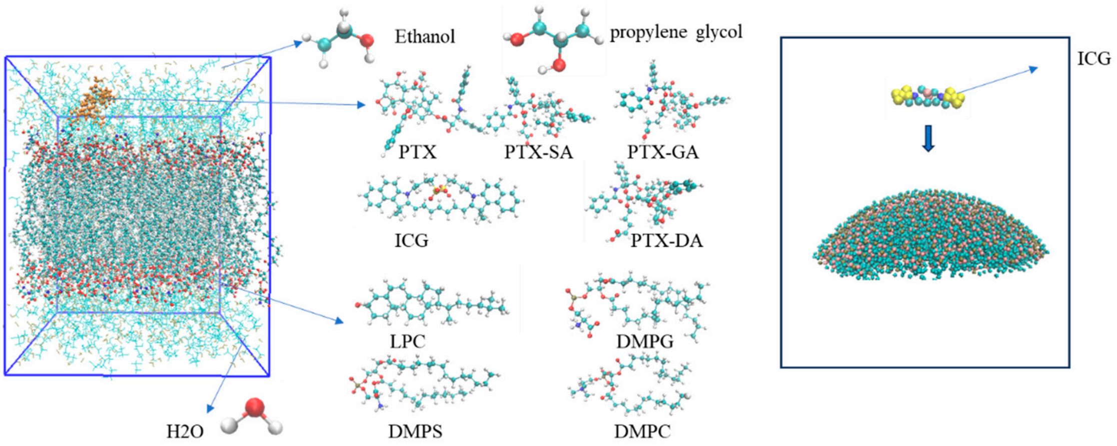

Figure S1. Schematic illustration of the simulation systems and molecular components used in this study.

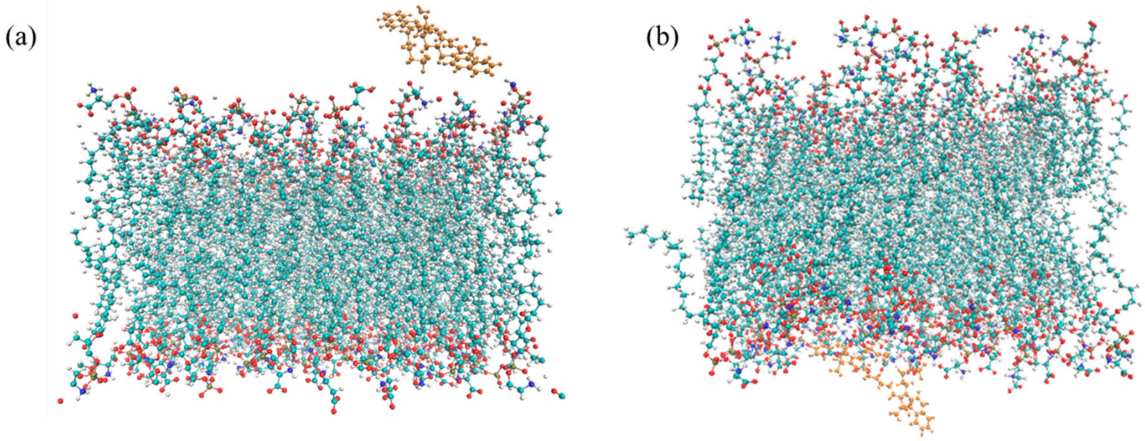

Figure S2. Representative initial and final configurations of an atomistic bilayer system used in this study.

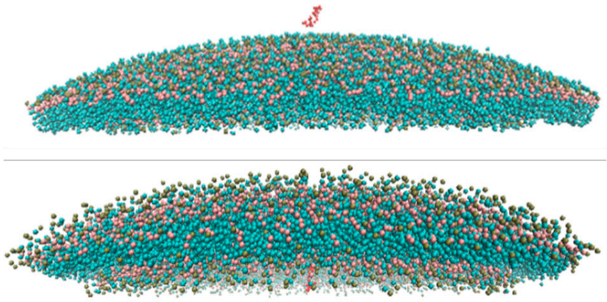

Figure S3. Representative initial and final configurations of the coarse-grained membrane model used for particle-size analysis. The model represents a local membrane region with curvature corresponding to a vesicle of the indicated equivalent diameter.

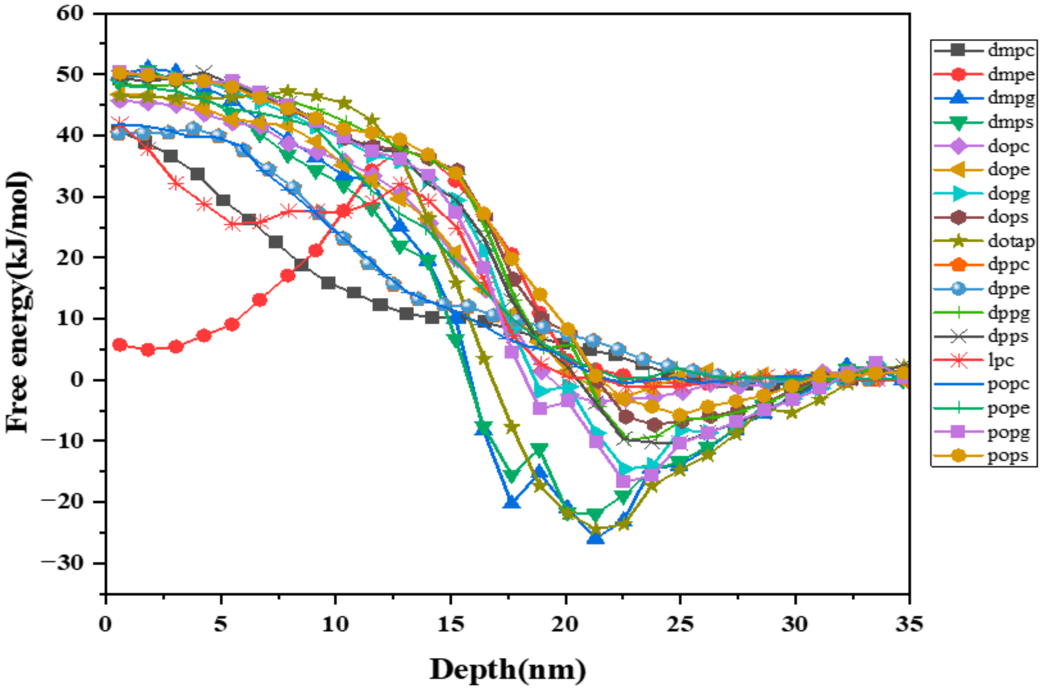

Figure S4. System enthalpy distribution at corresponding depth positions.

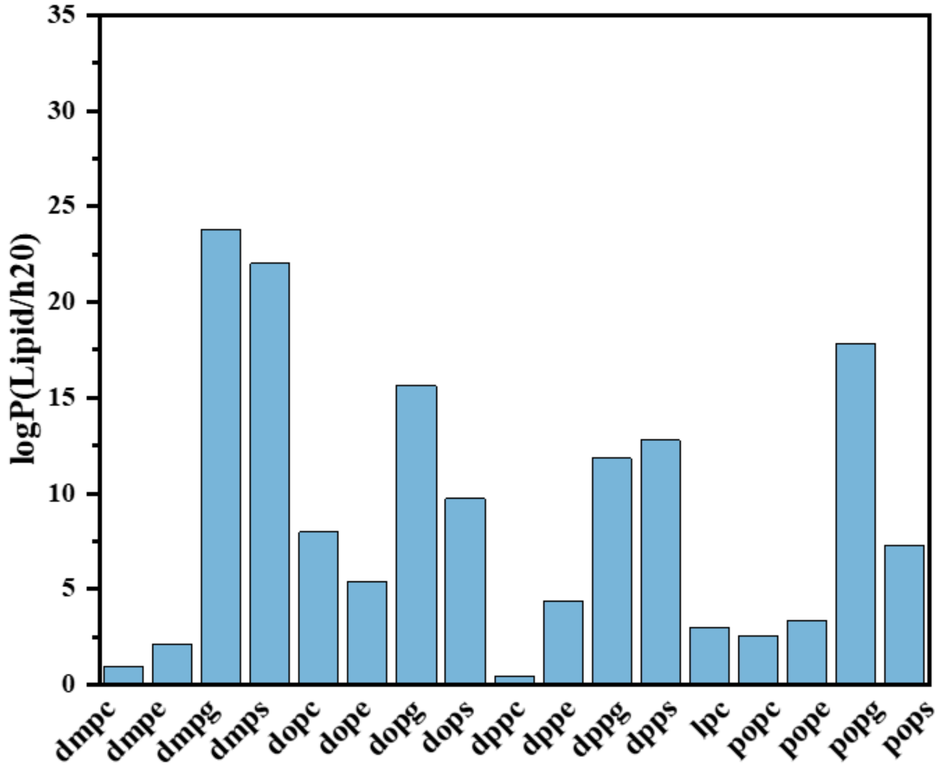

Figure S5. Partition coefficient (logP) of ICG in different lipid/water systems.

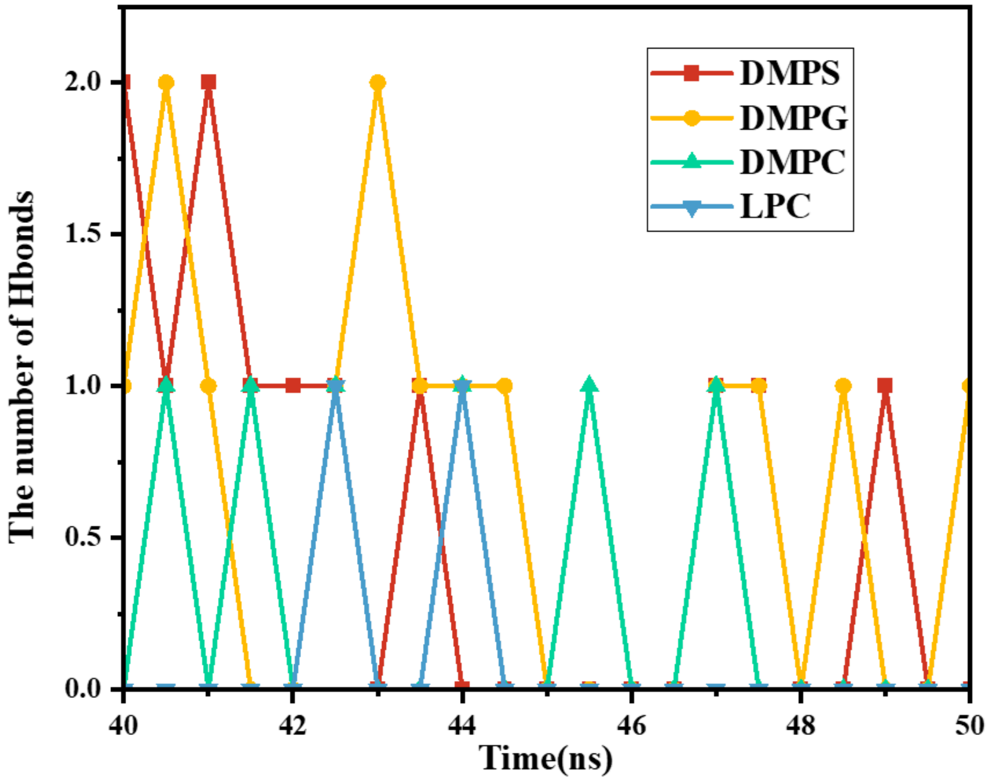

Figure S6. The number of H-bonds between DMPS and ICG.

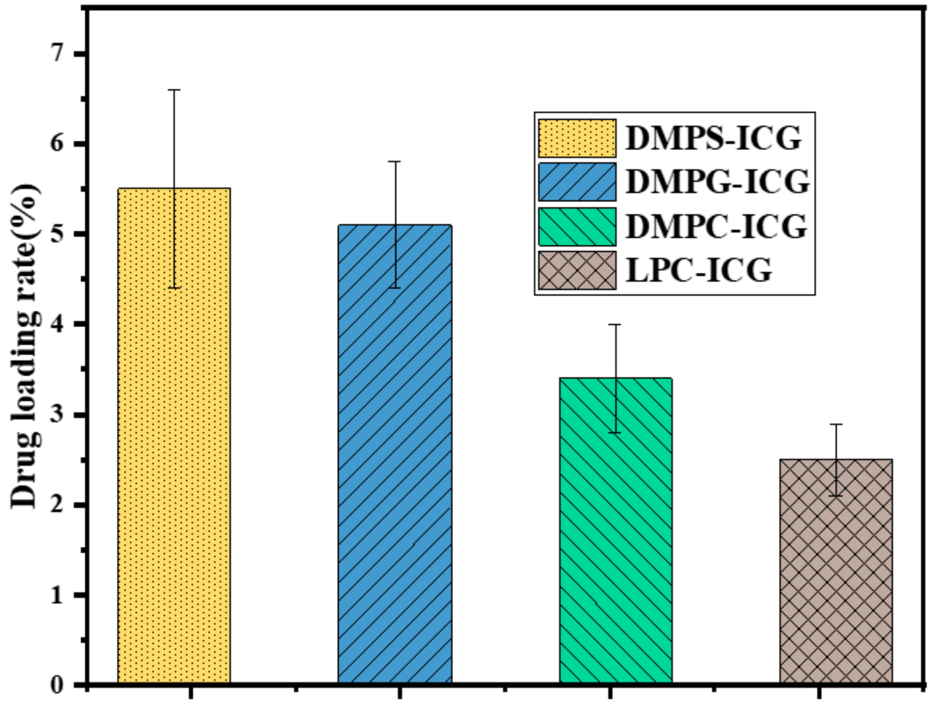

Figure S7. The drug-loading rate of ICG.

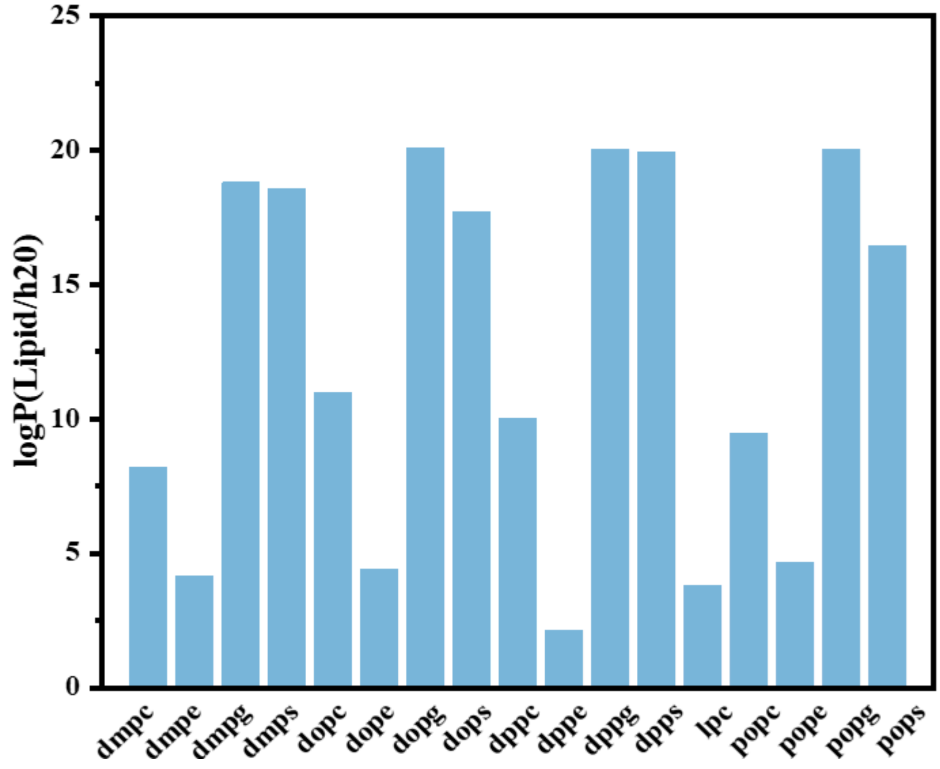

Figure S8. Partition coefficient (logP) of PTX in different lipid/water systems.

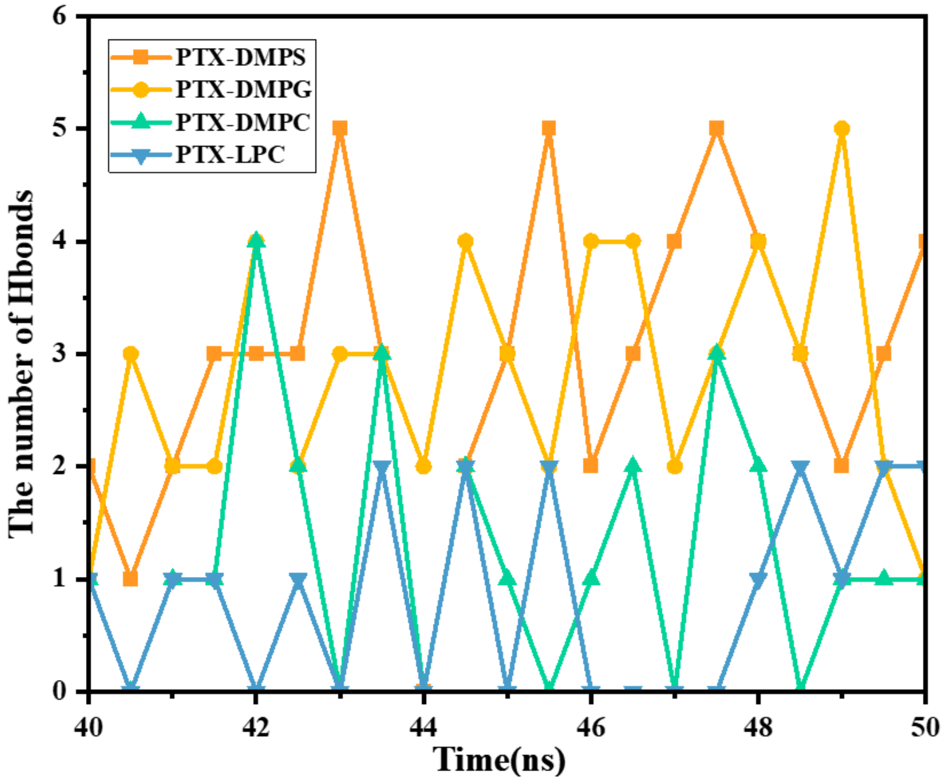

Figure S9. The number of H-bonds between DMPS and PTX.

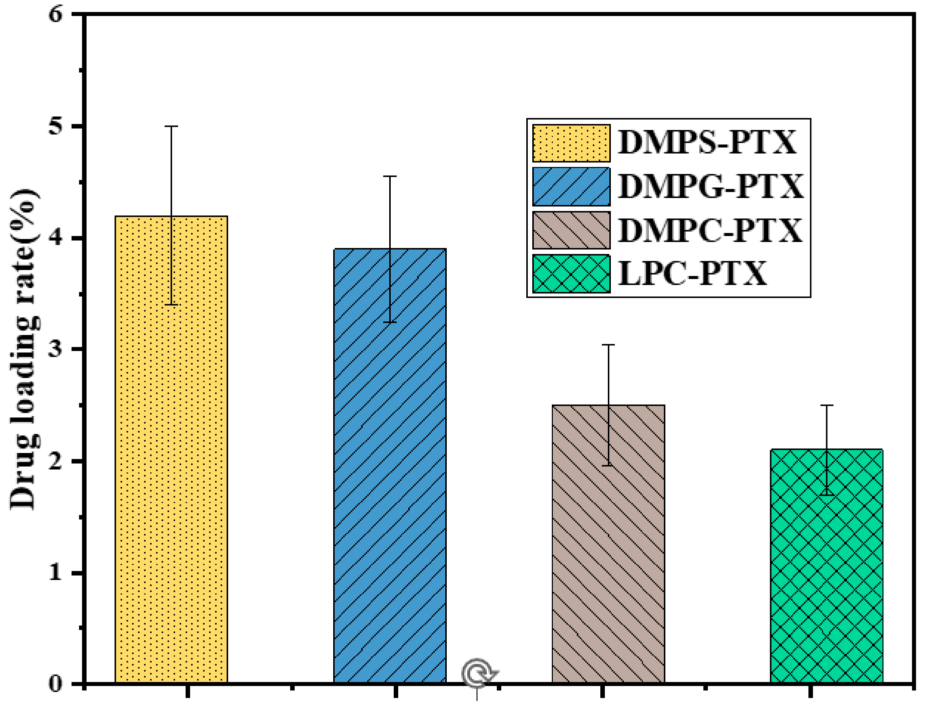

Figure S10. The drug-loading rate of PTX.

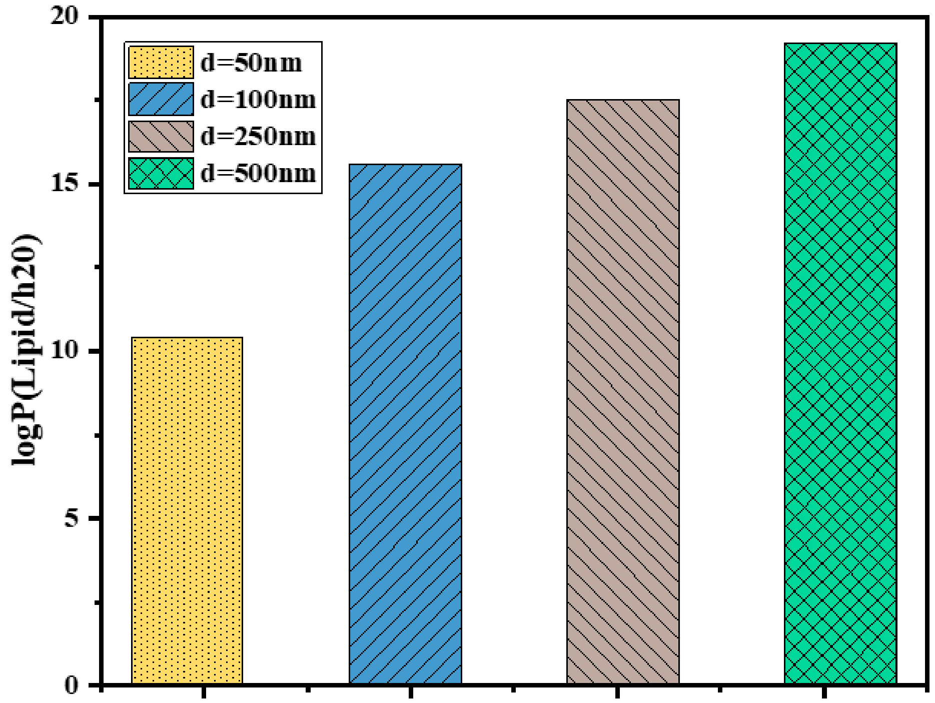

Figure S11. Partition coefficient (logP) of PTX.

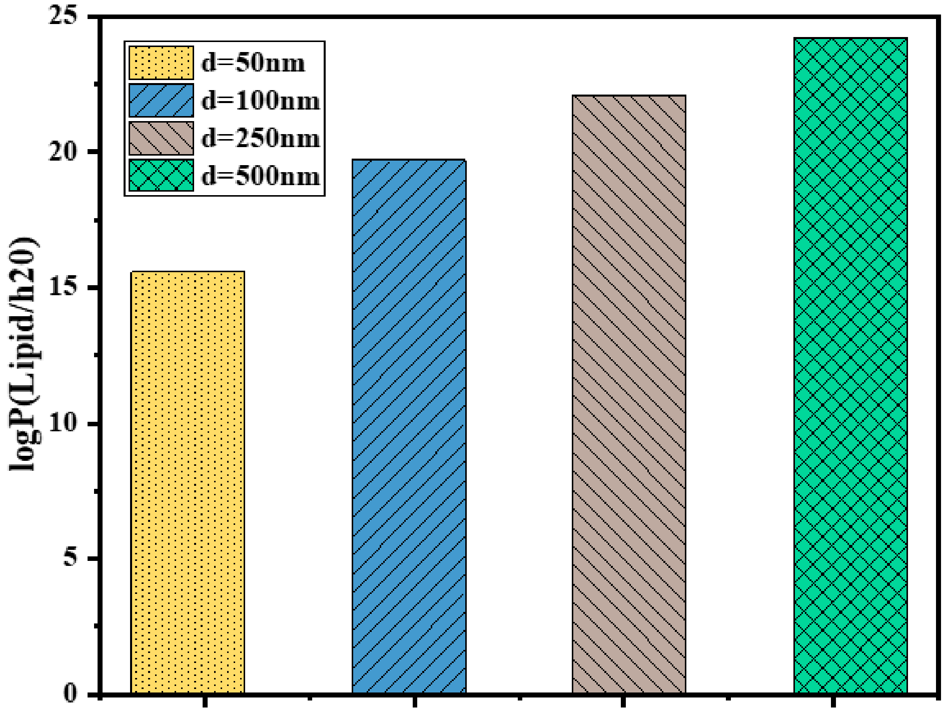

Figure S12. Partition coefficient (logP) of ICG.

Table S1. Molecular compositions of the atomistic bilayer systems used in this study.

System	Lipid type	Drug	Number of lipid molecules	Number of drug molecules	Number of water molecules	Number of Na+ ions	Number of Cl- ions	Box size (nm^3)
**DMPS-ICG**	**DMPS**	**ICG**	**124**	**1**	**3521**	**147**	**23**	**6 x 6 x6**
**DMPG-ICG**	**DMPG**	**ICG**	**124**	**1**	**3529**	**147**	**23**	**6 x 6 x6**
**DMPC-ICG**	**DMPC**	**ICG**	**124**	**1**	**3533**	**23**	**23**	**6 x 6 x6**
**LPC-ICG**	**LPC**	**ICG**	**124**	**1**	**3645**	**23**	**23**	**6 x 6 x6**
**DMPS-PTX**	**DMPS**	**PTX**	**124**	**1**	**3457**	**147**	**23**	**6 x 6 x6**
**DMPG-PTX**	**DMPG**	**PTX**	**124**	**1**	**3463**	**147**	**23**	**6 x 6 x6**
**DMPC-PTX**	**DMPC**	**PTX**	**124**	**1**	**3469**	**23**	**23**	**6 x 6 x6**
**LPC-PTX**	**LPC**	**PTX**	**124**	**1**	**3637**	**23**	**23**	**6 x 6 x6**
**DMPS-PTX-SA**	**DMPS**	**PTX-SA**	**124**	**1**	**3456**	**147**	**23**	**6 x 6 x6**
**DMPS-PTX-GA**	**DMPS**	**PTX-GA**	**124**	**1**	**3452**	**147**	**23**	**6 x 6 x6**
**DMPS-PTX-DA**	**DMPS**	**PTX-DA**	**124**	**1**	**3439**	**147**	**23**	**6 x 6 x6**

Table S2. Molecular compositions of the coarse-grained membrane models used for particle size analysis.

System	Vesicle diameter (nm)	Drug	Number of lipid molecules	Number of drug molecules	Number of water beads	Number of ions
DMPS-ICG-50	50	ICG	771	1	75547	771
DMPS-ICG-100	100	ICG	784	1	74821	784
DMPS-ICG-250	250	ICG	794	1	73801	794
DMPS-ICG-500	500	ICG	807	1	74075	807
DMPS-PTX-50	50	PTX	771	1	75544	771
DMPS-PTX-100	100	PTX	784	1	74819	784
DMPS-PTX-250	250	PTX	794	1	73800	794
DMPS-PTX-500	500	PTX	807	1	74071	807

Table S3. Molecular compositions of the atomistic solvent-containing systems used for solvent-effect analysis.

System	Lipid type	Drug	Solvent type	Solvent fraction	Number of lipid molecules	Number of drug molecules	Number of water molecules	Number of solvent molecules	Number of Na+ ions	Number of Cl- ions
ICG-H2O	DMPS	ICG	None	0%	124	10	3521	0	147	23
ICG-EtOH-20	DMPS	ICG	Ethanol	20%	124	10	3099	105	147	23
ICG-EtOH-40	DMPS	ICG	Ethanol	40%	124	10	2513	357	147	23

ICG-EtOH-60	DMPS	ICG	Ethanol	60%	124	10	1776	650	147	23
ICG-EtOH-80	DMPS	ICG	Ethanol	80%	124	10	1380	851	147	23
ICG-EtOH-100	DMPS	ICG	Ethanol	100%	124	10	0	1050	147	23
PTX-H2O	DMPS	PTX	None	0%	124	10	3457	0	147	23
PTX-EtOH-20	DMPS	PTX	Ethanol	20%	124	10	3054	100	147	23
PTX-EtOH-40	DMPS	PTX	Ethanol	40%	124	10	2488	357	147	23
PTX-EtOH-60	DMPS	PTX	Ethanol	60%	124	10	1708	638	147	23
PTX-EtOH-80	DMPS	PTX	Ethanol	80%	124	10	1391	818	147	23
PTX-EtOH-100	DMPS	PTX	Ethanol	100%	124	10	0	1049	147	23
ICG-PG-20	DMPS	ICG	Propylene glycol	20%	124	10	3194	80	147	23
ICG-PG-40	DMPS	ICG	Propylene glycol	40%	124	10	2505	250	147	23
ICG-PG-60	DMPS	ICG	Propylene glycol	60%	124	10	2027	379	147	23

ICG-PG-80	DMPS	ICG	Propylene glycol	80%	124	10	1695	472	147	23
ICG-PG-100	DMPS	ICG	Propylene glycol	100%	124	10	0	608	147	23
PTX-PG-20	DMPS	PTX	Propylene glycol	20%	124	10	3150	80	147	23
PTX-PG-40	DMPS	PTX	Propylene glycol	40%	124	10	2459	250	147	23
PTX-PG-60	DMPS	PTX	Propylene glycol	60%	124	10	1881	419	147	23
PTX-PG-80	DMPS	PTX	Propylene glycol	80%	124	10	1433	563	147	23
PTX-PG-100	DMPS	PTX	Propylene glycol	100%	124	10	0	597	147	23
